# Clear Cell Carcinoma of the Uterine Cervix in a 14-Year-Old Girl: A Case Report

**DOI:** 10.7759/cureus.77060

**Published:** 2025-01-07

**Authors:** Mayuko Motomura, Kazuhisa Hachisuga, Hiroshi Yagi, Hideaki Yahata, Kiyoko Kato

**Affiliations:** 1 Department of Obstetrics and Gynecology, Graduate School of Medical Sciences, Kyushu University, Fukuoka, JPN

**Keywords:** clear cell carcinoma, immune checkpoint inhibitor, immune-related adverse events, uterine cervix, young age

## Abstract

Clear cell carcinoma of the uterine cervix is a rare subtype. We report the case of a 14-year-old girl who presented with abnormal genital bleeding, a 7 cm mass arising from the uterine cervix, and swollen right inguinal lymph nodes. Histological and immunohistochemical examinations showed clear cell carcinoma. We performed a modified-radical hysterectomy, bilateral salpingo-oophorectomy, right inguinal lymph node biopsy, bilateral pelvic lymph node biopsy, and para-aortic lymph node biopsy, and diagnosed uterine cervical cancer stage IVB (clear cell carcinoma, pT2bN2M1). We administered paclitaxel-carboplatin+bevacizumab+pembrolizumab as adjuvant chemotherapy. Pembrolizumab is an immune checkpoint inhibitor associated with characteristic adverse events called immune-related adverse events (irAEs). In this case, arthritis and autoimmune pancreatitis were observed as irAEs during the treatment, but the patient was able to continue treatment. There is limited clinical experience with immune checkpoint inhibitors for pediatric patients. When remission is achieved, a better long-term prognosis is expected than in adults, so it is necessary to follow up with attention to late-onset irAEs.

## Introduction

Clear cell carcinoma of the uterine cervix (CCCC) is a rare histological type, which accounts for only 3-4% of uterine cervical adenocarcinomas [1]. Although most uterine cervical adenocarcinomas are associated with human papillomavirus (HPV), the HPV-positive rate of CCCC was reported to be lower than that of uterine cervical adenocarcinoma, the usual type [2]. According to previous reports, morbidity of CCCC shows a bimodal distribution with peaks at the ages of 26 and 71 years old [3] and the median age of patients with sporadic CCCC is 51 years [4]. Uterine cervical cancer is a rare cancer in childhood and adolescence, but adenocarcinoma is the predominant histologic type in childhood and adolescent cervical cancer [5]. In addition, CCCC at a young age has previously been reported in the context of intrauterine exposure to diethylstilbestrol (DES), a synthetic estrogen preparation [6]. The mean age of cases of CCCC due to DES exposure was reported to be 19 years old. We report a case of CCCC diagnosed at the age of 14 years without a history of DES exposure, with a discussion of the literature.

## Case presentation

A 14-year-old virgin girl had menarche from the age of 11. She consulted a local doctor because of continuous vaginal bleeding for about one month. A mass was found in her vagina. Therefore, she was referred to our hospital for detailed examination and treatment.

**Figure 1:**
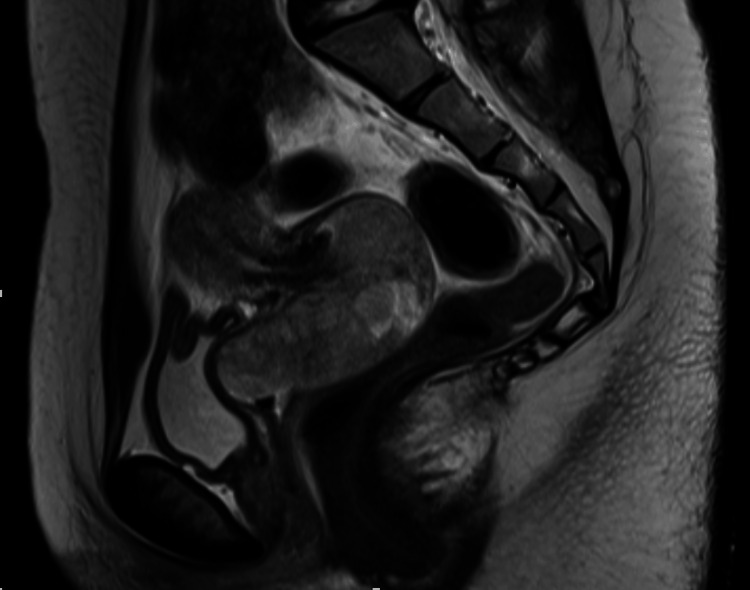
The representative image of MRI Contrast-enhanced T2-weighted MRI reveals a 68 × 37 mm mass originating from the uterine cervix and protruding into the vagina. No evidence of bladder or rectal involvement is observed.

**Figure 2:**
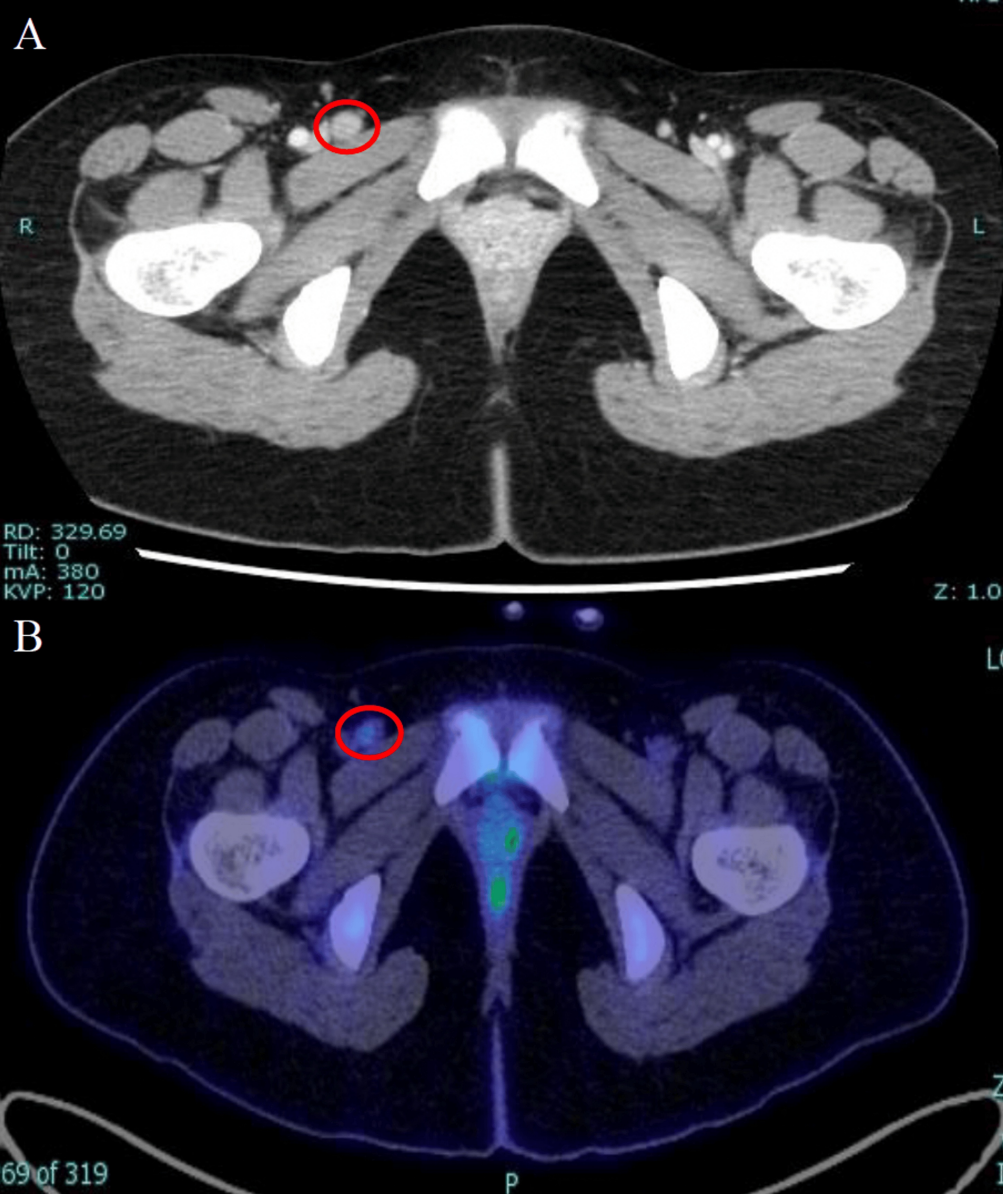
The representative images of CT and PET-CT (A) Contrast-enhanced CT shows mild enlargement of the right inguinal lymph nodes. (B) PET-CT demonstrates FDG accumulation in the same area (SUVmax 1.9). No evidence of distant metastasis is observed. CT: computed tomography; PET-CT: positron emission tomography-computed tomography; FDG: fluorodeoxyglucose; SUVmax: maximum standardized uptake value

On the physical examination, the right inguinal lymph nodes were swollen and external genitalia had a normal appearance. The ultrasound result showed a 63 x 44 mm mass in the vagina and thickening of the endometrium was not observed. Cervical histology was diagnosed with adenocarcinoma. HPV genotyping test was negative. The representative diagnostic images are shown in Figures 1-2. Pelvic contrast-enhanced MRI showed a 68 x 37 mm mass protruding into the vagina arising from the uterine cervix. There was no obvious rectal or bladder invasion. Contrast-enhanced CT showed enlarged bilateral pelvic lymph nodes and enlarged right inguinal lymph nodes. Positron emission tomography-computed tomography (PET-CT) showed mild fluorodeoxyglucose (FDG) accumulation in the right inguinal lymph nodes (SUVmax 1.9), which suggested local metastasis. There were no findings suggestive of distant metastasis. Based on these findings, we diagnosed adenocarcinoma of the uterine cervix, stage ⅣB (cT2a2N1M1).

**Figure 3:**
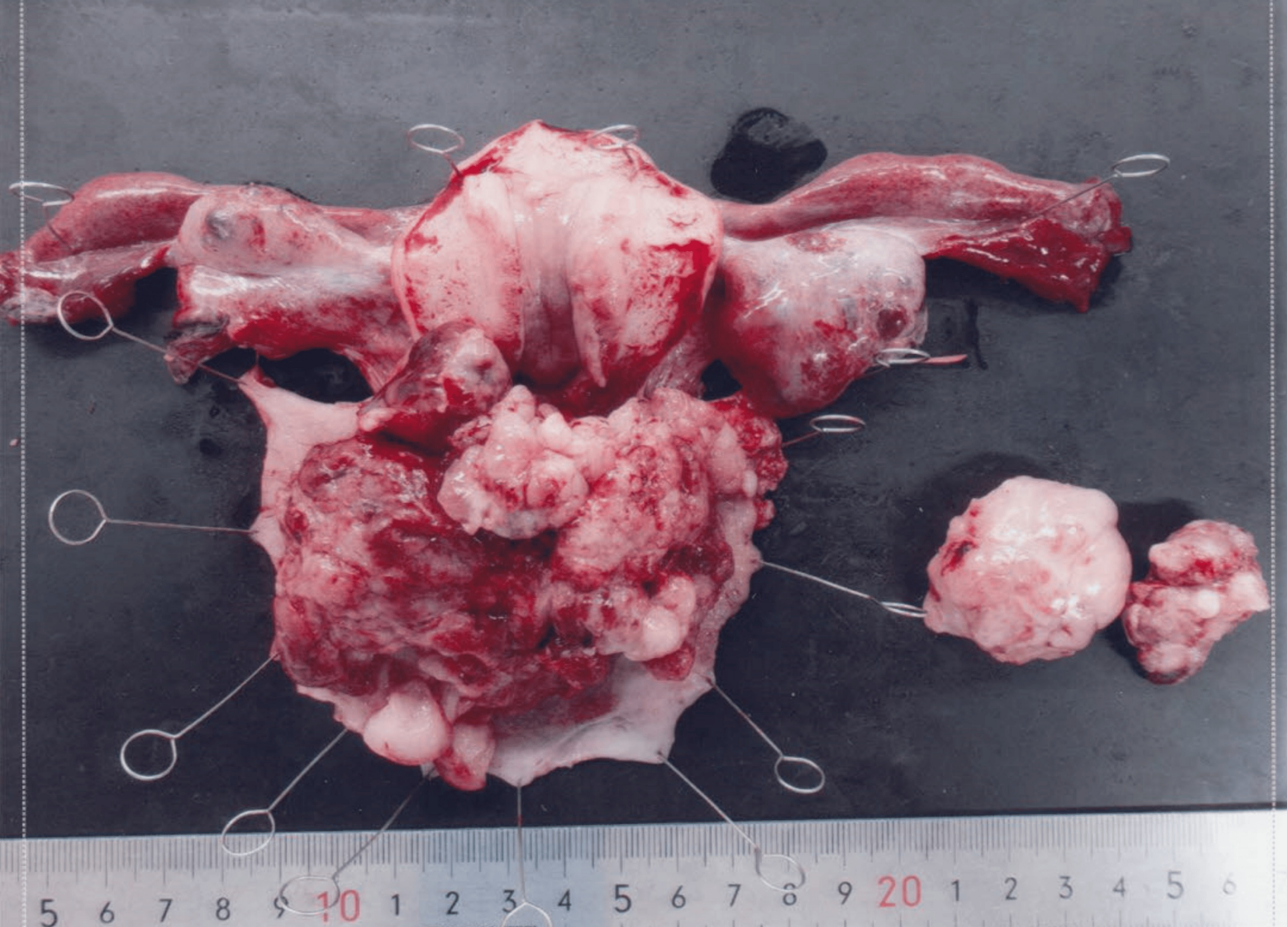
Macroscopic findings of the resected specimen An 8 × 6 cm fragile mass arising from the uterine cervix was observed. The bilateral adnexa appeared normal. A pelvic lymph node measuring up to 3 cm was also noted.

One month after diagnosis, we performed a modified-radical hysterectomy, bilateral salpingo-oophorectomy, right inguinal lymph node biopsy, bilateral pelvic lymph node biopsy, and para-aortic lymph node biopsy. Macroscopically, there was an 8 x 6 cm, large, easily hemorrhagic, and fragile mass arising from the uterine cervix (Figure 3). The bilateral adnexa was normal in appearance.

**Figure 4:**
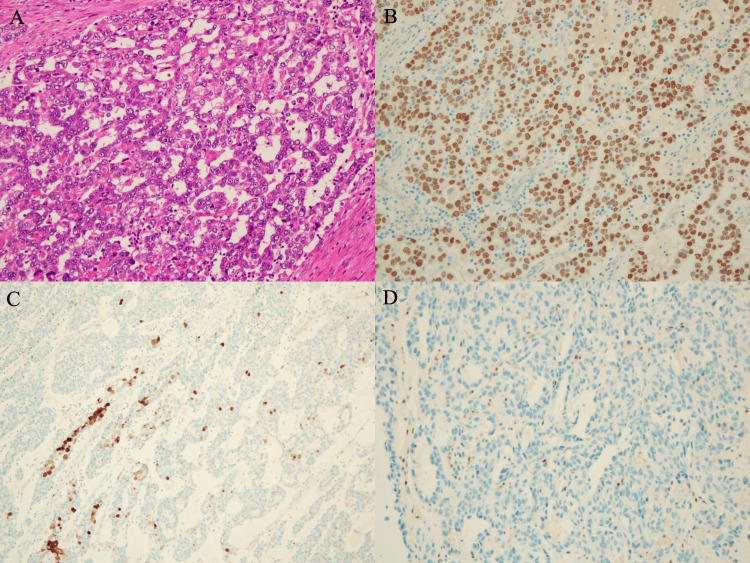
The representative images of HE and immunohistochemical staining (A) Hematoxylin and eosin (HE) staining: Tumor cells exhibit severe nuclear atypia and clear or eosinophilic cytoplasm, arranged in tubulocystic, irregular, glandular, cord-like, and solid patterns. (B) Immunohistochemical staining: HNF1β is diffusely positive in the tumor cells. (C) p16 shows partial positivity in the tumor cells. (D) Immunohistochemical staining: Estrogen receptor (ER) is negative in the tumor cells.

The representative images of hematoxylin and eosin (HE) and immunohistochemical staining are shown in Figure 4. Microscopically, the tumor was composed of severe nuclear atypia and clear or eosinophilic cytoplasm, arranged in a tubulocystic, irregular glandular, cord-like, and solid pattern. In addition, substantial vascular invasion was observed. Immunohistochemical staining showed diffuse positivity for HNF1β, partial positivity for p16, and negativity for estrogen receptor (ER). Based on pathological examination, the histologic type was diagnosed with CCCC. In addition, the pathological examination revealed deep stromal invasion (13/14 mm), negative for resection margins, positive for left cardinal ligament invasion, positive for bilateral pelvic lymph node metastasis, positive for left para-aortic lymph node metastasis, and positive for right deep inguinal lymph node metastasis. According to these findings, we determined that the postoperative diagnosis was CCCC, stage IVB (clear cell carcinoma, pT2bN2M1).

The first course of paclitaxel-carboplatin (TC) chemotherapy as postoperative adjuvant chemotherapy was administered approximately one month after surgery. From the second course, bevacizumab was added to TC chemotherapy. Since the combined positive score (CPS) exceeded 1, pembrolizumab was added from the third course. Twenty-six months have passed since the surgery without recurrence, and the patient is currently on maintenance therapy with bevacizumab and pembrolizumab.

## Discussion

CCCC is a rare histological type, which accounts for only 3-4% of uterine cervix adenocarcinomas [1]. CCCC is defined as an adenocarcinoma composed mainly of pallid or hobnail cells, showing a full, tubular/cystic, or papillary structure. It has a histological appearance similar to that of clear cell carcinoma arising in the endometrium, ovary, or vagina [1].

Although the median age of patients with sporadic CCCC is 51 years [4], Herbst reported that a history of fetal exposure to the synthetic estrogen DES is associated with the development of CCCC in young women [6]. Therefore, DES use during pregnancy was banned by the U.S. Food and Drug Administration (FDA) in 1971. Meanwhile, Hanselaar et al. reported that the age of onset of CCCC has a bimodal distribution with peaks at 26 and 71 years of age, regardless of a history of DES exposure. They speculated that this may be caused by changes in the hormonal environment during menarche and menopause, external factors other than DES, and genetic factors [3].

The relationship between CCCC and HPV infection is unclear. Pirog et al. studied the HPV-positive rate by histologic type in uterine cervical adenocarcinoma and reported that this rate was 71.8% in uterine cervical adenocarcinoma, the usual type, but 20% in clear cell carcinoma [2]. Currently, uterine cervical adenocarcinoma is classified as HPV-independent and HPV-associated, while CCCC is classified as HPV-independent [1].

Uterine cervical cancer is a rare cancer in childhood and adolescence, but adenocarcinoma is the predominant histologic type in childhood and adolescent cervical cancer [5]. In the literature, 28 cases of HPV-independent CCCC without a history of DES exposure that occurred at younger than 20 years of age have been previously reported, as shown in Table 1. Although the most common first symptom of CCCC is abnormal genital bleeding, such cases are sometimes difficult to diagnose in children and adolescents. Therefore, they are often misdiagnosed as precocious puberty or anovulatory bleeding.

**Table 1:** Summary of uterine cervical clear cell carcinoma cases under 20 years of age without prior DES exposure A total of 28 cases of HPV-independent uterine cervical clear cell carcinoma (CCCC) without a history of diethylstilbestrol (DES) exposure in patients under 20 years of age have been previously reported. FIGO: International Federation of Gynecology and Obstetrics

Report (Author and Years of Publication)	Age (years)	FIGO stage
Choi et al., 2013 [5]	15	IIA
Noller et al., 1974 [7]	7	IA
	10	IIB
	13	IB
	14	IIA
Wesolowski and Adelson, 1997 [8]	8	IB
Seki et al., 2003 [9]	18	IB2
Ding et al., 2004 [10]	19	IB2
Abu-Rustum et al., 2005 [11]	6	IB1
	8	IB1
Ahrens et al., 2005 [12]	6	IB1
Chan et al., 2008 [13]	14	IIIA
Yabushita et al., 2008 [14]	17	IB1
Lester et al., 2010 [15]	6	IB1
Singh et al., 2011 [16]	13	IB1
Ansari et al., 2012 [17]	14	IIIA
Jiang et al., 2014 [18]	19	IIA2
	20	IB1
Baykara et al., 2014 [19]	14	IB2
	16	IB1
Andi Asri et al., 2016 [20]	10	IB2
Arora et al., 2017 [21]	1	I
Tantitamit et al., 2017 [22]	19	IB1
Su et al., 2020 [23]	6	IIA1
Levinson et al., 2020 [24]	15	relapsed
Zhang et al., 2021 [25]	19	IIIB
Liu et al., 2021 [26]	12	IIIC1
Bujor et al., 2022 [27]	14	IIIC1

With regard to treatment, CCCC is generally considered to be resistant to cytotoxic chemotherapy such as platinum-based drugs and has a poor prognosis [28]. Reich et al. reported that the five-year survival rate was 67% for stages IB-IIB CCCC, which tended to be lower than those for squamous cell carcinoma (80%) and other cervical adenocarcinomas (77%), although the differences were not significant [29].

The treatment guidelines for uterine cervical cancer, 2022 edition, issued by the Japan Society of Gynecologic Oncology states that the standard treatment for stage I-II cervical cancer is radical hysterectomy or concurrent chemoradiotherapy, but a retrospective study reported that surgical therapy had a superior prognosis in cervical adenocarcinoma [30].

In this case, although in stage IVB, we performed surgery because it was considered difficult to achieve local control by only radiation therapy and chemotherapy due to the large size of the tumor. In addition, we administered TC+bevacizumab+pembrolizumab therapy as adjuvant chemotherapy.

Pembrolizumab is an immune checkpoint inhibitor approved for treating recurrent or advanced cervical cancer under Japanese national health insurance in September 2022. It is a human monoclonal antibody directed against PD-1, which negatively regulates T-cell functions by disrupting the binding between PD-L1/PD-L2 and PD-1. The KEYNOTE-826 trial showed significant prolongation in progression-free survival (PFS) and overall survival (OS) in patients with advanced or recurrent cervical cancer treated with pembrolizumab in combination with chemotherapy compared with chemotherapy only. Subgroup analyses were also performed on the efficacy of pembrolizumab by PD-L1 expression status for the populations with CPS<1, 1≤CPS<10, and CPS≥10. In these analyses, the CPS<1 population was defined as negative and the other two populations were defined as positive. All of these populations showed longer OS and PFS periods in the pembrolizumab combination group than those in the chemotherapy group. However, the CPS-negative population tended to have smaller OS and PFS prolongation than the CPS-positive population [31]. Of the 617 patients enrolled in the KEYNOTE-826 trial (446 squamous cell carcinoma, 140 adenocarcinoma, 29 adenosquamous carcinoma, and 2 other), the PD-L1-positive (CPS≥1) rate was 88.8% [31]. Zong et al. presented the rates of PD-L1 positivity by histological type of uterine cervical cancer and reported that 91% (91/100) of squamous cell carcinomas were PD-L1-positive, compared with 22% (11/50) of clear cell carcinomas [32]. Meanwhile, in this case, the patient with CPS≥1 was expected to respond to pembrolizumab, so she was administered TC+bevacizumab+pembrolizumab therapy. In addition, it has been reported that CPS may be predictive of persistent clinical response to immune checkpoint inhibitors off therapy, which is an important point in pediatric CCCC patients [33].

Immune checkpoint inhibitors such as pembrolizumab are associated with characteristic adverse events called immune-related adverse events (irAEs). Geoerger et al. described adverse events associated with the use of immune checkpoint inhibitors in children. They reported that 28 (18%) of 154 patients treated with pembrolizumab in the KEYNOTE-051 trial had irAEs [34]. The most common adverse events were hypothyroidism in 13 patients (8%), followed by hyperthyroidism in six patients (4%), pneumonia in three patients, thyroiditis in two patients, colitis in two patients, adrenal insufficiency in one patient, and serious skin reaction in one patient [34]. In that trial, pembrolizumab was well-tolerated and showed a therapeutic response that was as good as that of adults. However, there is insufficient data regarding late-onset irAEs with the use of immune checkpoint inhibitors in pediatric patients, and further data accumulation is needed.

Although there is limited experience with immune checkpoint inhibitors for CCCC in children, Levinson et al. reported a case of recurrent CCCC diagnosed at 15 years of age and treated with nivolumab, a PD-L1 inhibitor, which resulted in remission [24].

## Conclusions

This paper reports the case of a 14-year-old patient with stage IVB CCCC who was diagnosed with abnormal genital bleeding. It is important not to prematurely diagnose a hormonal disturbance, but to consider the possibility of malignant tumors, even in pediatric patients.

As for treatment, she was treated with TC+bevacizumab+pembrolizumab as postoperative adjuvant chemotherapy. CCCC tends to have a lower rate of PD-L1 positivity than uterine cervical squamous cell carcinoma. However, the CPS of this case was ≥1, and higher CPS is associated with prolonged remission. Therefore, it was decided to add bevacizumab and pembrolizumab to the conventional cytotoxic chemotherapy, but the differences in CPS between pediatric and adult CCCC need to be investigated in the future. With regard to adverse events, arthritis and autoimmune pancreatitis were observed as irAEs during the course of treatment, but the patient was able to continue treatment. There is limited clinical experience with immune checkpoint inhibitors for pediatric cervical cancer, and when remission is achieved, a better long-term prognosis is expected than in adults, so it is necessary to follow up with attention to late-onset irAEs.
